# Supplementary material for: The Effect of a Comprehensive, Interdisciplinary Medication Review on Quality of Life and Medication Use in Community Dwelling Older People with Polypharmacy
Source: J Clin Med. 2021 Feb 5;10(4):600. doi: 10.3390/jcm10040600 (PMC7915595; doi:10.3390/jcm10040600)
Supplement: Supplementary file 1 [file jcm-10-00600-s001.pdf]

Journal of
*Clinical Medicine*

MDPI

*Supplemtmental*

# The Effect of a Comprehensive, Interdisciplinary Medication Review on Quality of Life and Medication Use in Community Dwelling Older People with Polypharmacy

**Donna Bosch-Lenders [1], Jesse Jansen [1], Henri E.J.H. (Jelle) Stoffers [1], Bjorn Winkens [2], Karin Aretz [3], Mascha Twellaar [1], Jos M.G.A. Schols [4], Paul-Hugo M. van der Kuy [5], J. André Knottnerus [1] and Marjan van den Akker [1,6,7,*]**

[1] Department of Family Medicine, Care and Public Health Research Institute (CAHPRI), Maastricht University, 6200 MD, Maastricht, The Netherlands; donna.lenders@maastrichtuniversity.nl (D.B.-L.); jesse.jansen@maastrichtuniversity.nl (J.J.); jelle.stoffers@maastrichtuniversity.nl (H.E.J.H.S.); mascha.twellaar@maastrichtuniversity.nl (M.T.); andre.knottnerus@maastrichtuniversity.nl (J.A.K.)
[2] Department of Methodology and Statistics, Care and Public Health Research Institute (CAHPRI), Maastricht University, 6200 MD, Maastricht, The Netherlands; bjorn.winkens@maastrichtuniversity.nl
[3] MEMIC, center for data and information management, Faculty of Health, Medicine and Life Sciences, Maastricht University, 6200 MD, Maastricht, The Netherlands; Karin.Aretz@maastrichtuniversity.nl
[4] Department of Health Services Research, Care and Public Health Research Institute (CAHPRI), Maastricht University, 6200 MD, Maastricht, The Netherlands; jos.schols@maastrichtuniversity.nl
[5] Department of Hospital Pharmacy, Erasmus MC, University Medical Center Rotterdam, 3015 GD Rotterdam, The Netherlands; h.vanderkuy@erasmusmc.nl (H.v.d.K.)
[6] Institute of General Practice, Goethe University, 60590, Frankfurt am Main, Germany
[7] Academic Centre of General Practice, Department of Public Health and Primary Care, KU Leuven, 3000, Leuven, Belgium
* Correspondence: m.vandenakker@allgemeinmedizin.uni-frankfurt.de; Tel.: +49-6301-80454

**Abstract: Background:** We conducted a comprehensive medication review at the patients' home, using data from electronic patient records, and with input from relevant specialists, general practitioners and pharmacists formulated and implemented recommendations to optimize medication use in patients aged 60+ years with polypharmacy. We evaluated the effect of this medication review on quality of life (QoL) and medication use. **Methods:** Cluster randomized controlled trial (stepped wedge), randomly assigning general practices to one of three consecutive steps. Patients received usual care until the intervention was implemented. Primary outcome was QoL (SF-36 and EQ-5D); secondary outcomes were medication changes, medication adherence and (instrumental) activities of daily living (ADL, iADL) which were measured at baseline, and around 6- and 12-months post intervention. **Results:** Twenty-four general practices included 360 women and 410 men with an average age of 75 years (SD 7.5). A positive effect on SF-36 mental health (estimated mean was stable in the intervention, but decreased in the control condition with −6.1, $p = 0.009$,) was found with a reduced number of medications at follow-up compared to the control condition. No significant effects were found on other QoL subscales, ADL, iADL or medication adherence. **Conclusion:** The medication review prevented decrease of mental health (SF36), with no significant effects on other outcome measures, apart from a reduction in the number of prescribed medications.

**Keywords:** polypharmacy; primary care; medication review; cluster-randomized controlled trial; general practitioner; pharmacist; patient; home visit

**Citation:** Bosch-Lenders, D.; Jansen, J.; Stoffers, H.E.J.H.; Winkens, B.; Aretz, K.; Twellaar, M.; Schols, J.M.G.A.; van der Kuy, P.H.M.; Knottnerus, J.A.; van den Akker, M,. The Effect of a Comprehensive, Interdisciplinary Medication Review on Quality of Life and Medication Use in Community Dwelling Older People with Polypharmacy. *J. Clin. Med.* **2021**, *10*, 600. https://doi.org/10.3390/jcm10040600

Received: 23 December 2020
Accepted: 2 February 2021
Published: 5 February 2021

**Publisher's Note:** MDPI stays neutral with regard to jurisdictional claims in published maps and institutional affiliations.

**Copyright:** © 2021 by the authors. Licensee MDPI, Basel, Switzerland. This article is an open access article distributed under the terms and conditions of the Creative Commons Attribution (CC BY) license (http://creativecommons.org/licenses/by/4.0/).

# 1. Polypharmacy Intervention Limburg (PIL) Study–Training of the Participants

## 1.1. Training of the General Practitioners (GPs) and the Community Pharmacists

Before each of the three periods in which practices would enter the intervention phase, we organized training sessions on medication review for participating GPs and community pharmacists who were to start with the intervention in the forthcoming period. We invited collaborating GPs and pharmacists to participate together in the same training session (they could choose between two dates).

We organized training sessions (two per period, six in total) in a central location in the region, from 6 pm to 9.30 pm. The program included background information on polypharmacy and the PIL study, and a workshop “practice of medication reviews”. In the workshop, the PIL intervention was practiced, using three different paper cases of patients with polypharmacy (small-group work, wrap up and learning points in a plenary session). A GP and a pharmacist, members of the PIL study team, moderated the training sessions. Accreditation points for continuous medical education (CME) were awarded upon completion of the training session. We provided the participants with a handout and background literature on pharmacology in the elderly and frequently occurring interactions.

The training session's learning goal was to teach GPs and pharmacists how they can assess a patient with polypharmacy according to a stepwise approach to revise the medication list of the patient in a more appropriate direction. GPs and pharmacists learned to share, interpret and integrate information and knowledge they each have about the patient; this included medical diagnoses, recent laboratory results and medication issued by the pharmacist. Particular attention was paid to indications of prescribed medication, possible side effects, and interactions, taking into account the information acquired by the practice nurse during a visit to the patient’s home.

GPs and pharmacists had to formulate a preliminary medication recommendation according to the steps described in Figure 2 in the main text, under #4.

Based on the comprehensive information obtained in the previous steps (see Figure 2 in the main text), pharmacists and GPs formulated a preliminary integrated medication recommendation tailored to the patient's specific health profile as follows:

- Medication (prescribed and over-the-counter) is categorized in seven groups: cardiovascular, diabetes mellitus, gastrointestinal tract, pulmonary diseases, psychotropic drugs, pain medication and “other”.
- Medication is checked for indications as recorded in the GP's EPR and in correspondence with the medical specialists involved in the care for the patient.
- Side effects and adverse effects as reported by the patient are listed.
- Drug–drug interactions (as reported in the GP’s or pharmacist’s EPR or by the patient) are listed, as well as known contraindications, interactions between drugs or diseases, pseudo-double medication, and appropriate dosage using the Beers List, and the STOPP/START criteria.
- Finally, the way medication is administered is evaluated (e.g., dose aerosol with a spacer instead of a powder inhaler; simplification of dosing when possible, e.g., one 40 mg tablet once a day instead of 20 mg tablets twice a day etc.) and instructions and prescriptions are adjusted when needed.

A translation of the slides we used during the training sessions is presented below.

### Medication Review: a Systematic Approach

- All medications
- All indications
  - *Consider non-pharmacological intervention*
  - *Stop medication if indication is not clear*
- Always address side effects (known or unknown)
- *'Start low, go slow'*

### Medication review in 6 steps

1. Inventory of all possible medication
   - (GP's EMR vs. pharmacy vs. patient at home)
2. Classification in medication groups
3. Indications (and contra indications)?
   - under treatment and over treatment
4. Adverse side effects?
5. Interactions?
6. Mode of administration and dosage?

### Step 1/6: Inventory of all possible medication

- GP's electronic medical record (EMR)

Check/compare with:

- Pharmacy 's information system
- Patient (e.g. home visit by practice nurse (PN))

### Step 2/6: Classification in medication groups

(*to facilitate comparison with list of diagnoses*)

1. Cardiovascular
2. Diabetes
3. Gastro-intestinal
4. Pulmonary: Asthma/COPD
5. Psychotropic drugs
6. Analgesics
7. Rest

### Step 3/6: Indications (and contra indications)?

*(under treatment and over treatment)*

- **Under** treatment: should you **START** a drug?
  - *Given the list of diagnoses , what drugs are missing? **
- **Over** treatment: **STOP** or reduce medication?
  - *No indication: what is superfluous?*
    *(or: complete the list of diagnoses!)*
  - *Contra-indications: potential harm*?*
    - *Depending on diagnoses in list*
    - *Older patients: independent of diagnoses in list*
    - *Renal function?*

* E.g.: **Beers list; STOPP/START criteria.** See literature references at end of handout

### Step 4/6: Adverse side effects?

- All symptoms mentioned as such by the patient , are taken into account
- Consider potential known side effects yourself
- Check EMR for previously registered side effects
- Important factors to assess whether it is an adverse effect:
  - *Known as a side effect*
  - *Time relation start medication and occurrence of phenomenon*
  - *Disappearance of phenomenon if drug is stopped*
  - *(and reappearance if drug is started again)*
  - *No alternative explanation*

### Step 5/6: Interactions?

- Given the medication status, are there any relevant risks relating to interactions between various drugs?

### Step 6/6: Dosage & Mode of administration?

- Caution with medications with narrow therapeutic range
- Adjust dose to renal function – eGFR - (or severely impaired) hepatic function *
- Frequency of dosing
- Other types of inhalers
- Other administration options?
- Tools, like such as blister, baxter, dosette box

### *1.2. Training of the Practice Nurses (PN) (Home Visits)*

The role of the practice nurse (PN) in the PIL study was threefold:

- She made telephone calls inviting patients to participate; if the patient accepted the invitation, the PN made an appointment for a home visit.
- She extracted data from the practice's electronic medical record system (problem list, medication, correspondence with specialists, results of recent laboratory tests and if these were older than three months, had them re-recorded)
- She made home visits to the participating patients.

Before each of the three periods in which practices would enter the intervention phase, we organized training sessions for practice nurses of the practices that would start with the intervention in the forthcoming period. They could choose between two dates.

We organized training sessions (two per period, six in total) at the Department of Family Medicine of Maastricht University, in Maastricht, from 1.15 pm to 4.15 pm. The program included background information on polypharmacy and the PIL study, and a workshop on how to perform the home visits. In the workshop, we familiarized the PNs with the questionnaire (checklist) they would use when visiting the patients at home, and we practiced the interview with them. After an example, the participants, in small groups, using simulated patients, did two role-plays. Members of the PIL study team moderated the training sessions. We provided the participants with a handout.

We explained the home visit's primary goal: to get a complete overview of the patient's medication use, prescribed and "over-the-counter" (OTC) medication. We provided them with a copy of the questionnaire (checklist) and explained what information they should collect and how.

**Box S1: The content of the training on completing the questionnaire on medication use.**

1. Measure height, weight, blood pressure
2. Interview on medication use:

- WHAT medicines does the patient use? Prescribed medication, over-the-counter drugs (including creams and ointments, homoeopathic medicines and herbal remedies.
- HOW does the patient use this medication? Special attention was paid to adherence (being taken according to the dosage instruction, being taken in the right way (e.g., inhalers, eye drops; the PNs were instructed to ask patients to demonstrate how they do it).
- Does the patient KNOW for what reason (s)he is taking each medication? Which INDICATION does the patient mention?
- Does the patient experience SIDE EFFECTS?
- Is there any medication that the patient is NOT taking (anymore)?
- Are there relevant details concerning DAILY FUNCTIONING? Limitations concerning taking medication (e.g., rheumatism, visual impairment, forgetfulness, etc.)?
- Is any (informal) CARER involved?
- Are SPECIALISTS involved? (name, discipline hospital, last and upcoming visit)

**Box S2: The content of the instruction on interview skills.**

- The interviewer is in control: you want to have these questions answered as well and complete as possible; this requires a direct, active questioning attitude.
- Ask open questions.
- Listen well: note what the patient tells you and do not interpret, do not note your personal associations or conclusions; pay attention to verbal and non-verbal cues and then continue to ask further.
- Summarize often.
- Reflect: provide feedback to the interviewee, who can then respond further, e.g., when you notice hesitation or doubt; or when vague words are used.
- Examples of a few questions: Which tablet would you omit first and why? Which tablets would you like to recommend to others and which never? If you had to explain to someone why you are taking which tablets, how would you do that? How satisfied are you with the current situation, what needs to be done to improve it? Etc.

### *1.3. Role-Play in Small Groups*

We used simulated patients, played by study team members. We used 2–3 different case studies. The role descriptions included detailed medical, psychological and social background information, including information about medication use and instructions on responding to questions from the interviewing PN.

The three case descriptions differed in used drugs and locations where the patient “stored” the medication. We did this to raise the PN's awareness of the wide variety of options that patients can demonstrate in this regard.

**Box S3: Examples of places where patients may keep their medication.**

- Case 1: basket on the living room table, dresser drawer, in the kitchen, on the bedside table
- Case 2: shoebox on living room cupboard, medication on the living room table, on the bedside table, in their bathroom
- Case 3: the patient had a “Baxter”; therefore, a caregiver was involved. However, other medications, including OTC drugs, were managed by the patient herself, and were kept in the kitchen drawer and the bedside table.

### *1.4. Examples of Parts of the Checklist on Medication Use, Shown During the Training*

A. Prescribed medication (e.g., on 2. diabetes, 3. gastrointestinal tract; column titles: drug name, dosage as printed on the package, actual frequency by the patient, the reason for taking medication if different from the instruction on the package, adverse effects, indication for medication according to the patient)

| 2.Medicatie DM | | | | | |
|---|---|---|---|---|---|
| Naam medicament | Dosering | Frequentie inname | Andere inname dan voorschrift omdat | Bijwerkingen | Indicatie volgens patiënt |
| | | | | | |
| | | | | | |
| | | | | | |
| | | | | | |
| 3.Medicatie tractus digestivus | | | | | |

| Naam medicament | Dosering | Frequentie inname | Andere inname dan voorschrift omdat | Bijwerkingen | Indicatie volgens patiënt |
|---|---|---|---|---|---|
| | | | | | |
| | | | | | |
| | | | | | |
| | | | | | |
| | | | | | |

B. Example of how to complete the checklist on medication use (e.g., on 5. psychotropic drugs; column titles: see above)

| Medicatie psychofarmaca | | | | | |
|---|---|---|---|---|---|
| Naam medicament | Dosering | Frequentie inname | Andere inname dan voorschrift omdat | Bijwerkingen | Indicatie volgens patiënt |
| Brotizolam | 0,25 mg | 0,5 dd 1 | - | | voor het slapen |
| Mirtazipine | 15 mg | n.v.t.<br>0 of - | - | | ? |
| Seroquel | 50 mg | 1dd1 | - | | ? |
| | | | | | |

C. Over-the-Counter medication (drug name, dosage, actual frequency, description of use, e.g., when needed/when having a headache), adverse effects, indication according to the patient)

| Zelfzorgmedicatie | | | | | |
|---|---|---|---|---|---|
| Naam medicament | Dosering | Frequentie inname | Andere inname dan voorschrift omdat | Bijwerkingen | Indicatie volgens patiënt |
| | | | | | |
| | | | | | |
| | | | | | |

D. Information on specialists (name, discipline, hospital, last visit, upcoming visit)

| Behandeling Specialist | | | | | |
|---|---|---|---|---|---|
| Naam specialist | Specialisme | Ziekenhuis | Laatste controle | Volgende controle | opmerkingen |
| | | | | | |
| | | | | | |
| | | | | | |
| | | | | | |
